# Microorganism-Based Strategies for the Control of Cyanobacterial Blooms: A Review of Recent Progress

**DOI:** 10.3390/toxins17120604

**Published:** 2025-12-17

**Authors:** Wangle Zhang, Shiyuan Meng, Xiaoxu Wu, Hong Shen, Dongqin Wang, Tong Qiu, Weijie Li, Jiping Chen, Ling Li, Bingbing Liang, Mengdi Zhao, Xuwei Deng, Chi Zhou

**Affiliations:** 1 Hubei Water Resources Research Institute, Hubei Water Resources and Hydropower Science and Technology Promotion Center, Wuhan 430070, China; zhangwl@whu.edu.cn; 2 School of Chemistry, Chemical Engineering and Life Sciences, Wuhan University of Technology, Wuhan 430070, China; 3 Center for Eco-Environmental Science and Technology of Wuhan, Wuhan 430022, China; 4 Institute of Hydrobiology, Chinese Academy of Sciences, Wuhan 430072, China; 5 Wuhan Second Ship Design and Research Institute, Wuhan 430205, China; 6 University of Chinese Academy of Sciences, Beijing 100049, China; 7 School of Ecology and Environment, Tibet University, Lhasa 850012, China; 8 School of Ecology and Environment, Anhui Normal University, Wuhu 241000, China; 9 State Key Laboratory of Plateau Ecology and Agriculture, Qinghai University, Xining 810016, China

**Keywords:** algicidal microorganisms, cyanobacterial blooms, ecological safety, harmful algal bloom mitigation, microbial control

## Abstract

Cyanobacterial blooms, which are increasingly exacerbated by eutrophication and climate change, pose threats to ecosystems and public health. This paper systematically reviews recent advances in microbial intervention strategies for controlling cyanobacterial blooms. Current approaches primarily comprise direct lysis methods, indirect suppression methods, and integrated strategies. Direct algicide methods rapidly lyse cyanobacterial cells and degrade toxins, although their application is constrained by environmental sensitivity and host specificity. Indirect approaches offer sustainable preventive strategies by inhibiting cyanobacterial growth, yet require careful environmental management. Integrated methods combine microbial strategies with other technologies, enhancing both the efficiency and ecological safety of managing cyanobacterial blooms. While microbial strategies demonstrate significant potential, practical implementation faces challenges, including environmental adaptability, ecological safety, and regulatory frameworks. Future research should focus on integrating synthetic biology, intelligent delivery systems, and multi-omics technologies to achieve more effective and environmentally friendly management of cyanobacterial blooms.

## 1. Introduction

Cyanobacteria, commonly referred to as blue-green algae [1], are prokaryotic microorganisms that are widely distributed across aquatic environments, including freshwater, marine, and wetland ecosystems [2]. As an important component of these ecosystems, cyanobacteria contribute significantly to primary production and nutrient cycling [3]. However, under certain environmental conditions, cyanobacteria can undergo rapid proliferation and aggregation, resulting in cyanobacterial blooms [4]. These blooms typically form conspicuous blue-green or blue-brown scums at the water surface [5]. The impacts of cyanobacterial blooms are extensive and severe. Some cyanobacterial species produce cyanotoxins, such as microcystins, which pose significant health risks to humans by contaminating drinking water and aquatic food products [5,6]. Exposure to these toxins can cause liver and nervous system damage and has been linked to an increased risk of cancer [5]. Ecologically, cyanobacterial blooms lead to oxygen depletion in water bodies through the decomposition of accumulated biomass, often resulting in hypoxic or anoxic conditions that cause mortality among fish and shellfish [7]. Additionally, dense blooms reduce light penetration [8], inhibiting the growth and photosynthesis of submerged plants and thereby disrupting material cycling and energy flow throughout the aquatic ecosystem [9]. As a result, cyanobacterial blooms pose a serious threat to both global aquatic ecosystems and public health, highlighting the urgent need for effective management and control strategies.

Currently, three main approaches have been employed for controlling cyanobacterial blooms: physical, chemical, and biological methods. Physical methods, such as mechanical harvesting and artificial mixing, can temporarily reduce cyanobacterial biomass and are often used in smaller water bodies or for emergency intervention. However, these methods are costly, labour-intensive, and largely ineffective for large-scale or sustained bloom management [10]. Chemical controls, including the application of algicides like copper sulfate, can rapidly suppress cyanobacterial populations [11]. Nonetheless, these agents may also harm non-target aquatic organisms, disrupt ecological balance, and leave harmful residues that can result in secondary pollution [9]. By contrast, microbial remediation is attracting increasing attention due to its high efficiency, specificity and environmental compatibility [12]. This approach uses microorganisms such as algicidal bacteria, actinomycetes, and bacteriophages that have the capacity to inhibit or lyse cyanobacteria through the secretion of bioactive compounds, competition for resources, or direct parasitism [13]. Microbial remediation technology has the additional benefit of not only enabling water bodies to self-purify but also avoiding other damage to native aquatic communities. However, it is important to note that while microbial and natural compound-based methods are generally considered more environmentally friendly, they are not entirely without risk. Some natural compounds produced by microorganisms, such as cyanotoxins and mycotoxins, can pose ecological or health hazards. Therefore, a comprehensive evaluation of potential environmental impacts is essential when developing and deploying these biological control strategies. Nevertheless, the microbial intervention strategies examined in this review, including direct lysis, indirect inhibition, and integrated approaches, all constitute in situ direct control methods. These aim to suppress cyanobacterial blooms through the direct action of microorganisms or by modulating the microenvironment. Control of cyanobacterial blooms also encompasses indirect control pathways, centred on limiting bloom-driving factors such as nutrient inputs and releases like nitrogen and phosphorus. While not the primary focus of this review, such strategies constitute an essential component of integrated bloom management.

Despite promising progress, microbial remediation technologies continue to face challenges in both development and practical application. Firstly, the precise mechanisms by which microorganisms control cyanobacteria remain incompletely elucidated, and the regulatory pathways governing these interactions require further investigation. Furthermore, the environmental adaptability, stability, and persistence of microbial formulations across diverse natural water bodies represent critical issues for ensuring sustained algal bloom control, as certain microorganisms exhibit limited survival rates and efficacy under field conditions. Concurrently, the diversity of aquatic characteristics further impacts the effectiveness of microbial interventions, hindering their widespread adoption. Finally, other research continues to explore solutions addressing these challenges, with a particular focus on enhancing the applicability and robustness of microbial remediation techniques across diverse aquatic environments.

In this review, a comprehensive assessment of microbial approaches to cyanobacterial bloom control is provided. First, microbial interventions are categorised based on their direct and indirect mechanisms of action, as well as their use in integrated management strategies in combination with other technologies. Subsequently, recent research progress is summarised, practical case studies are highlighted, and the effectiveness and limitations of current methods are evaluated. Finally, existing knowledge gaps are discussed, and directions for future research are proposed to boost the development and implementation of microbial methods for effective cyanobacterial bloom mitigation.

## 2. Microorganisms Directly Lysing Cyanobacteria

### 2.1. Algicidal Bacteria

Algicidal bacteria refer to bacteria that can inhibit the growth of algae or kill algae, thereby lysing algal cells through direct or indirect means [14]. The reported algicidal bacteria and their primary target cyanobacteria are listed in Table 1.

**Figure 1:**
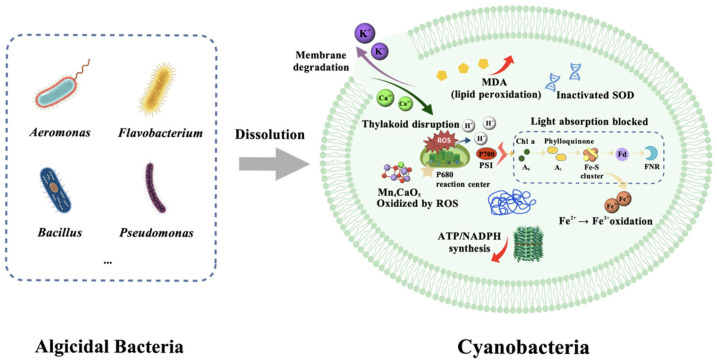
Schematic diagram of the mechanism of action of algicidal bacteria on cyanobacteria.

Figure 1 shows the key processes by which algicidal bacteria inhibit or lyse cyanobacterial cells through various pathways, including membrane damage, photosynthesis interference, and oxidative stress induction [46,47].

*Aeromonas* species primarily control cyanobacterial blooms through direct contact. Park et al. [15] reported that when *A. bestiarum* strain HYD0802-MK36 comes into direct contact with *Microcystis aeruginosa*, it induces cyanobacterial cell death; conversely, its cell-free culture filtrate exhibits no algicidal activity against cyanobacteria. The algicidal substance is primarily located within the cytoplasm. Cell fractionation experiments revealed that the cytoplasmic fraction exhibited the highest specific activity (60 units/mg), whereas periplasmic and membrane fractions showed lower specific activities (−6.3 to 4.4 units/mg), suggesting the algicidal agent may be an intracellular enzyme. The algicidal substance likely resembles protease enzymes, requiring direct contact to disrupt cyanobacterial cell structures. Inactivation upon heating or treatment with proteinase K further supports its proteinaceous nature.

Furthermore, *A. bestiarum* strain HYD0802-MK36 exhibits significant algicidal activity solely against the target cyanobacterium *M. aeruginosa* (91%), displaying extremely low activity (−143% to 20%) against 14 non-target algae (including green algae, diatoms, and dinoflagellates), confirming its status as a highly specific algicidal bacterium. This high specificity avoids harm to non-target algae, minimises ecological side effects, and renders it more suitable for targeted control of cyanobacterial blooms.

As for *Bacillus*, in the treatment involving *Bacillus* sp. strain AK3 immobilised on biochar and the denitrifying bacterium *Alcaligenes* sp. strain M3, cyanobacterial density decreased from 600,000 cells/mL to 80,000 cells/mL, while chlorophyll-a (Chla) concentration fell from 85.7 μg/L to 42.8 μg/L [18]. *Bacillus* sp. strain AK3 may secrete specific enzymes or metabolites that disrupt cyanobacterial cell walls or membranes, directly causing cell lysis and death. When the cell density of *Bacillus licheniformis* strain Sp34 reaches at least 1.35 × 10^5^ CFU/mL, it can eliminate the DCM4 strain of *M. aeruginosa* and other harmful algae in Dianchi Lake, such as *M. wesenbergii* and *Phormidium* sp. This strain exerts its effect by releasing algicidal substances exhibiting excellent thermal stability (−20 °C to 121 °C) and pH tolerance (pH 3–11) [21]. Its algicidal activity shows a significant positive correlation (*p* < 0.001) with algal growth conditions, demonstrating higher efficacy when algal growth is more vigorous.

Li et al. [48] focused on the algicidal mechanism of *Raoultella* sp. strain S1 against *M. aeruginosa*, conducting an in-depth investigation through Tandem Mass Tag (TMT) quantitative proteomics and other methodologies. The cell-free supernatant exhibited pronounced flocculation and inactivation effects on algal cells at low (1 × 10^6^ cell/mL) and medium (2.7 × 10^6^ cell/mL) concentrations, achieving 72-h kill rates of 96.23% and 92.46%, respectively, whilst efficacy markedly diminished at high concentrations (5.4 × 10^6^ cell/mL), yielding a killing rate of merely 37.21%. At low and medium concentrations, algal cells exhibited more pronounced antioxidant defence responses, characterised by elevated malondialdehyde (MDA) levels and significantly enhanced activity of antioxidant enzymes, including superoxide dismutase (SOD), catalase (CAT), and peroxidase (POD). In contrast, oxidative stress responses were relatively weaker in high-concentration algal cells. Photosynthetic efficiency and relative electron transport rate (rETR) declined substantially within 24 h. Transmission electron microscopy revealed disruption of thylakoid membranes and cellular structures. TMT proteomics analysis revealed differential protein changes during flocculation and lysis phases. The core mechanism of lysis in the supernatant was damage to the photosynthetic system, with impaired conversion of light energy into chemical energy, downregulation of carbohydrate metabolism proteins, and suppression of DNA replication and repair, leading to cyanobacterial cell apoptosis. This mechanism rapidly aggregates and precipitates algal cells, reducing their capture and utilisation of light energy. However, when algal cell concentrations are high, both kill rates and flocculation rates decline considerably, potentially limiting their effectiveness in managing large-scale cyanobacterial blooms.

*Pseudomonas fluorescens* strain SK09 can directly act upon algal cells by secreting metabolites with algicidal activity, such as certain enzymes or secondary metabolites. These substances disrupt the cell wall or cell membrane structure, leading to lysis and death of the algal cells. In mesocosm experiments conducted at water temperatures ranging from 5 to 15 °C, the maximum algal lysis effect (MAE) observed after inoculation with SK09 reached −17.4% to 83.2%, validating its algicidal potential under mesophilic and cold conditions [49]. Furthermore, when SK09 effectively lyses algae, it releases inorganic nutrients (e.g., DIN, DIP, DS_i_). For instance, in closed microcosms, DIP concentrations significantly increased after SK09 inoculation. Carrier immobilisation partially mitigates nutrient release through adsorption, balancing ecological impacts.

However, in this study, in outdoor mesocosm experiments, when water temperatures dropped to 0.1–3.0 °C (corresponding to river surface freezing), SK09 exhibited MAE values of −5.92% and −1.67% against algal cells, showing no significant difference from the control group (*p* > 0.05). This indicates that SK09 is unable to exert algicidal effects near freezing temperatures. In direct application experiments on the Nakdong River, reduced interaction time between bacteria and algae due to river flow resulted in no significant difference in algal cell abundance at three sampling points compared to the control group. Algicidal efficacy is inferior under low-temperature conditions near freezing and in flowing water environments, like rivers.

Bauer and Forchhammer [22] have documented the algal lysis processes of *Bdellovibrio* and *Myxococcus* across multiple dimensions. *Bdellovibrio* are obligate endosymbiotic predators that exclusively lyse Gram-negative bacteria, with prey ranges varying between strains. Their two-stage predation strategy involves a predatory phase where chemotaxis guides prey acquisition, followed by secretion of enzymes to form invasion channels. During the periplasmic growth phase, cellular components are degraded within the periplasmic space for reproduction. Prolonged growth culminates in host cell wall destruction by lytic enzymes, releasing progeny. Its genome encodes over 250 hydrolases, with metabolism relying on prey to compensate for nutritional deficiencies. Host-independent variants exist due to reduced Type IV pili formation. *B. bacteriovorus* lyses *Phormidium luridum*, while *B. exovorus*, an ectosymbiont predator, attaches to prey surfaces to consume cytoplasm. *Bdellovibrio* and Like Organisms (BALOs) strains can invade *M. aeruginosa* for internal lysis.

*Myxococcus xanthus*, a soil-dwelling Gram-negative bacillus, exhibits specific amino acid nutritional deficiencies requiring acquisition from prey, though it may also be saprophytic. It necessitates direct physical contact with prey, employing multiple synergistic lysis mechanisms against a broad range of hosts. The PCO_2_ strain of *M. xanthus* can capture and lyse *Phormidium luridum* via aqueous colonies.

In predatory modes, *Bdellovibrio* exhibits endosymbiosis while *M. xanthus* practises extracellular symbiosis; locomotion depends on chemotaxis and flagellar movement versus type IV pili and gliding, respectively; prey ranges encompass Gram-negative bacteria for the former and broader species for the latter; Metabolic characteristics comprise obligate predation or host-independent metabolism in the former, alongside facultative predation and saprophagy in the latter. Lysis mechanisms involve periplasmic enzymatic digestion and cell wall modification in the former, and collective secretion of hydrolytic enzymes in the latter. Both contribute to cyanobacterial population regulation, influencing aquatic ecological equilibrium. Nevertheless, high nitrate environments may suppress their activity, potentially promoting cyanobacterial blooms.

Liu et al. [42] reported the algicidal activity of *Serratia marcescens* strain BWL1001 against *M. aeruginosa*. This strain efficiently synthesises serratiamycin, exhibiting potent algicidal activity against harmful algae. *M. aeruginosa* was first cultured to a logarithmic phase at approximately 4 × 10^6^ cells/mL. Subsequently, serratiamycin at concentrations of 0.1, 0.5, 1.0, and 3.0 μg/L purified phycoerythrin, with untreated samples as controls. Cultivation proceeded for 48 h in TAP medium under a 12-h light/12-h dark cycle at 23 °C. Algal cell density was determined by measuring absorbance at 540 nm. Results demonstrated a dose-dependent relationship between phycoerythrin and its algicidal activity against *M. aeruginosa*, achieving a 91.1% algicidal rate at 3.0 μg/L, indicating potent algicidal efficacy. As a microbial metabolite, leuco-cinnabarite is naturally degradable, reducing the risk of aquatic pollution associated with chemical agents and aligning with green biocontrol requirements. However, concerns remain regarding whether its inhibition of algal cells may induce resistance evolution in algal species, or whether it could exert secondary effects on aquatic food chains. Furthermore, the precise mechanism of its algicidal action on algal cells—including specific targets and signal pathways affected—remains unclear.

For *Alcaligenes aquatilis*, its algicidal efficacy depends on the initial cell density of *M. aeruginosa*. When the initial *Microcystis* density was 5 × 10^6^ cells/mL, *A. aquatilis* strain F8 achieved an algicide rate of 88.45% within 72 h. At a density of 5 × 10^7^ cells/mL, the algicide rate decreased to 74.41%, outperforming most previously reported strains [17]. Transmission electron microscopy (TEM) revealed that the cell-free culture filtrate (CCF) of strain F8 disrupted *Microcystis* cell membranes, leading to the disappearance of photosynthetic lamellae and disruption of phycobiliproteins. Furthermore, sodium alginate encapsulation was employed to immobilise strain F8. However, the immobilised strain exhibited a lower algicidal rate (85.46% at 72 h) than the free-living strain (87.06%), attributed to the immobilisation matrix restricting bacterial activity. Adding wheat bran to the immobilisation matrix significantly enhanced the algicidal rate to 95.49%, representing an 8.83% improvement over the free-living strain. Acid-treated wheat bran, which degrades starch, cellulose, and vitamins, restored algicidal activity to the level of the immobilised control group. Conversely, alkaline-treated wheat bran, which primarily degrades proteins and lipids, had a lesser impact, suggesting vitamins may be the key component. Subsequently, adding a multivitamin complex to acid-treated wheat bran restored algicidal activity, while supplementing with individual vitamins such as E and B_2_ promoted bacterial growth, thereby enhancing algicidal activity. Starch and cellulose proved ineffective as carbon sources for strain F8, whereas vitamins within wheat bran were central to promoting its growth and algicidal activity. As an inexpensive agricultural by-product, wheat bran simultaneously supplies carbon, nitrogen, and vitamins to the bacteria. Its auxiliary immobilisation technique offers a low-cost, highly efficient solution for in situ management of cyanobacterial blooms, combining environmental benefits with economic viability.

Jeong et al. [33] reported that they isolated thirty bacterial strains from co-cultures of *Myriophyllum spicatum* and *M. aeruginosa*. Following screening, strain MS16 was identified as exhibiting significant algicidal activity against *M. aeruginosa*. 16S rRNA gene sequence analysis confirmed its identification as *Nocardioides convexus*.

The algicidal activity of strain MS16 against *M. aeruginosa* primarily occurs through direct physical attack. When treated with bacterial washings, algicidal rates reached 84% within one day and 98% within two days. Scanning electron microscopy revealed MS16 adhering to *M. aeruginosa* cell surfaces, causing structural disruption. Comparing algicidal effects between bacterial culture medium, bacterial wash solution, and cell-free filtrate demonstrated significantly higher activity in the wash solution and culture medium than in the filtrate, indicating MS16 primarily acts through direct contact rather than phytohormone secretion. Furthermore, MS16 exhibits selective inhibitory effects on *M. aeruginosa*, with no significant impact on other cyanobacteria or green algae, and even promotes the growth of certain green algae. Following MS16 treatment, gene expression related to photosynthesis (*rbc*L), oxidative stress defence (SOD, GSH), toxin synthesis (*mcy*A), and cellular repair (*fts*H, *rec*A) was significantly downregulated in *M. aeruginosa*, resulting in the loss of its growth and survival capacity.

MS16 exhibits high algicidal efficacy against *M. aeruginosa*, achieving 98% inhibition within a short timeframe and remaining effective even at high algal cell densities. Furthermore, it specifically targets certain cyanobacteria such as *M. aeruginosa*, exerting minimal impact on other algae and aquatic organisms, thereby contributing to the maintenance of ecosystem diversity. Although algal cell lysis during initial treatment releases microcystins, toxin concentrations decline rapidly thereafter, potentially indicating toxin degradation capacity that mitigates environmental hazards.

However, against natural populations of *M. aeruginosa* in water bodies, the algicidal efficacy of MS16 may be diminished by the protective mucilage layer surrounding the algal cells, necessitating longer exposure times or higher bacterial concentrations to achieve optimal results. When deploying MS16 on a large scale for *M. aeruginosa* bloom treatment, cultivation costs and application dosages must be carefully considered to ensure economic viability.

Li et al. [29] revealed that strain h10 exhibits highly effective algicidal activity against *M. aeruginosa* 7820. Identification confirmed its affiliation with the genus *Exiguobacterium*, and it was designated as *Exiguobacterium* sp. strain h10. Experiments demonstrated that the algicidal activity primarily emanated from the culture supernatant rather than the bacterial cells themselves, indicating indirect attack via secreted algicidal substances rather than direct contact. The algicidal molecule, with a molecular weight below 1000 Da, exhibited poor thermal and pH stability, suggesting a possible glycolipid mixture. It caused significant reduction in pigment content, such as Chla, disrupted algal cell structure and morphology, thereby inhibiting algal growth. Structurally, algal cells suffer severe damage to their structure and morphology, including cell wall and cell membrane injury, resulting in loss of cell integrity. Physiologically, Chla synthesis is inhibited, leading to decreased intracellular pigment content and impaired photosynthesis. This strain and its algicidal substance hold promise as a potential biocontrol agent for managing *M. aeruginosa* blooms, offering novel approaches and materials for the biological control of cyanobacterial blooms.

*Chryseobacterium* sp. exhibited significant algicidal activity against *M. aeruginosa* FACHB 905 under initial optimised conditions (30 °C, pH 7, bacterial concentration 1 × 10^8^ CFU/mL, *M. aeruginosa* concentration 6 × 10^5^ cells/mL), achieving an algicidal rate of 80% at 72 h [27]. This lysis exhibited marked time dependency: slight yellowing of the algal suspension commenced after 12 h of co-culture, with the lysis rate rising to 35% at 24 h and accelerating rapidly to 62% at 48 h. The lysis rate levelled off thereafter at 72 h. The 0.22 μm membrane-filtered culture group also demonstrated effective algal lysis, further confirming that this strain primarily exerts indirect lysis through secreted extracellular active substances rather than direct contact.

The group subjected to high-temperature sterilisation (121–123 °C for 20 min) retained high algal lysis activity, indicating that the secreted extracellular algolytic substances possess strong thermal stability and are not proteinaceous in nature. Algicidal activity was observed across the 4–37 °C range, with the highest lysis rate (80%) at 30 °C. Rates decreased significantly at 10 °C (32%) and 4 °C (18%). Effective algicidal activity persisted within the pH range of 5–9, peaking at pH 7 (80%). Increasing bacterial concentration from 1 × 10^6^ CFU/mL to 1 × 10^8^ CFU/mL elevated the algicidal rate from 28% to 80%; further increasing to 1 × 10^9^ CFU/mL only raised the rate to 82%, with the increase levelling off. When *M. aeruginosa* concentration rose from 6 × 10^5^ cells/mL to 6 × 10^7^ cells/mL, the algal lysis rate decreased from 80% to 44.4%, indicating significant inhibition of lysis efficacy by high algal density.

Physiologically, following the addition of the bacterial strain supernatant, Chla content exhibited a marked decline from day 1, falling to 330 mg/m^3^ by day 5. By day 7, the removal rate reached 59.37%, demonstrating a highly significant difference compared to the control group (*p* < 0.01). On day 1 of co-culture, malondialdehyde (MDA) accumulation in algal cells of the treatment group increased by 39.95% compared to the control group (*p* < 0.01), continuing to rise with prolonged culture duration, indicating severe disruption of algal cell membrane structures. Phosphatidylcholine (PC) fluorescence values exhibited a consistent downward trend parallel to Chla changes, confirming comprehensive destruction of the algal photosynthetic system during algal lysis.

Structurally, after 2 days of co-culture, algal cells exhibited deformation and aggregation; by day 4, partial cell rupture and leakage of contents occurred; by day 7, extensive cell disintegration resulted in debris-like precipitates in the culture medium. Processed algal cells exhibited significantly reduced absorption peak intensities at 1647 cm^−1^ (C-O bond), 2927 cm^−1^ (C-H bond), and 3437–3475 cm^−1^ (O-H bond). Characteristic minor absorption peaks emerged in the 2500–1700 cm^−1^ range, indicating destruction of polysaccharide and protein structures and cellular disintegration.

Wang et al. [34] investigated the algicidal mechanism of the algicidal bacterium *Paenibacillus* sp. strain SJ-73. Cultures of SJ-73, cell-free filtrates, and washed bacterial cells were co-cultured with *M. aeruginosa* PCC 7806. Algal efficiency was determined by measuring Chla content. The algicidal efficiencies of the culture and cell-free filtrate were 90.48% and 89.51%, respectively, whilst the washed bacterial cells exhibited no inhibitory effect. This indicates that SJ-73 exerts its algicidal action by secreting extracellular active substances rather than through direct contact with the algal cells. The secreted active substances cause cell lysis by disrupting the cell membrane system and cell wall of *Microcystis*. Subsequently, various algae (including cyanobacteria and green algae) were treated with 5% SJ-73 filtrate, with Chla content measured after 7 days. The algicidal efficiencies against *M. aeruginosa* PCC 7806, *Dolichospermum circinale* PCC 7120, and *Aphanizomenon flos-aquae* FACHB 1168 were found to be 83.97%, 71.35%, and 71.04%, respectively. Whereas the inhibition rates for *Chlorella vulgaris* FACHB 26 and *Anabaena oblique* FACHB 416 were merely 3.67% and 8.03%. Thus, SJ-73 exhibits potent algicidal activity against cyanobacteria (particularly *Microcystis*), with minimal impact on green algae, demonstrating marked host specificity. SJ-73 exhibits highly efficient algicidal activity against both unicellular and colonial *Microcystis*, though the process induces substantial release of microcystins (MCs), with higher concentrations of filtrate (10%) yielding more pronounced MCs release. Its algicidal efficacy exceeded 90% against both single-celled and colonised *Microcystis*, while also exhibiting significant inhibition against other cyanobacteria (e.g., *Anabaena*, *Synechococcus*), making it applicable for controlling large-scale multi-species blooms. However, MCs released during algicide action can exceed initial concentrations by over 20-fold, potentially posing threats to aquatic organisms and human health. SJ-73 alone can only remove *Microcystis* biomass without simultaneously degrading released MCs, necessitating additional toxin degradation methods like MlrA enzyme treatment, thereby increasing management steps and costs. Furthermore, while the 10% filtrate concentration demonstrated slightly higher algicidal efficacy (94.38%), MCs release increased by 24.5% compared to the 5% treatment group, indicating that high-dose application may exacerbate toxin contamination.

To sum up, to enhance the academic application of algicidal bacteria, future research should integrate proteomics and bioinformatics to identify differential surface proteins between *Microcystis* and non-target organisms, enabling the selection of ideal target molecules. By leveraging computer-aided design (CAD) and artificial intelligence (AI), binding protein domains may be engineered to target these molecules precisely. And with the designed genes synthesised through synthetic biology, integrating into naturally occurring *Microcystis*-infecting bacteriophages, creating precision-guided phages. Among the studied strains, *Nocardioides convexus* strain MS16 emerges as a particularly promising candidate due to its high specificity, rapid lysis, minimal ecological disruption, and potential toxin degradation capacity, which deserves further exploration for cyanobacteria bloom management.

### 2.2. Algicidal Fungi

Algicidal fungi are eukaryotic microorganisms that inhibit or lyse algal cells through the secretion of extracellular enzymes, oxidative metabolites, or secondary compounds that disrupt membrane integrity, photosynthetic electron transport, or cellular redox balance [50]. The fungi currently reported to possess highly potent algicidal activity are primarily concentrated within the genera *Phanerochaete*, *Trichoderma*, *Aspergillus*, and those producing photosensitive metabolites, such as *Cercospora* (which produces cercosporin). Their target algal species encompass typical harmful algal bloom species in both freshwater and marine environments, including cyanobacteria, green algae, cryptophytes, and dinoflagellates. Significant variations in algal species selectivity exist among different fungi. For instance, *T. citrinoviride* exhibits potent inhibition solely against *M. aeruginosa*, while showing no inhibitory effect and even mildly promoting growth in green algae and diatoms [51]. Conversely, *Phanerochaete chrysosporium* achieves highly efficient algicidal activity against three algal species: *Cryptomonas obovata*, *Oscillatoria* sp., and *Scenedesmus quadricauda*. Specifically, the mechanisms of action for algicidal fungi can be categorised into three types: direct physical effects, metabolite-mediated indirect effects, and dual effects driven by photosensitization.

#### 2.2.1. Direct Physical Contact and Lysis Mechanisms

Represented by *P. chrysosporium* [50] and *T. citrinoviride* [51], these fungi achieve algicidal effects through physical adsorption, enveloping algal cells, and directly disrupting their structure.

For *P. chrysosporium*, scanning electron microscopy (SEM) revealed that its hyphae initially adhere to the algal cell surface. Subsequently, the cell membrane integrity of *C. obovata*, *Oscillatoria* sp., and *S. quadricauda* significantly deteriorates. Flow cytometry (FCM) analysis indicates that the proportion of dead/severely damaged cells reaches 82.4–86.5% (compared to only 1.5–2.4% in the control group). Transmission electron microscopy (TEM) revealed cell wall rupture, protoplasmic leakage, and complete disintegration of thylakoid structures. Concurrently, fungal biomass exhibited marked augmentation. Following 48-h co-culture with *S. quadricauda*, fungal mycelial dry weight increased from an initial 0.056 ± 0.005 g to 0.527 ± 0.067 g (*p* < 0.001), suggesting fungal nutrient acquisition through algal cell degradation [50]. Following 1 day of co-culture between *T. citrinoviride* and *M. aeruginosa*, mycelial enveloping traces appeared on algal cell surfaces, with complete cell rupture observed after 2 days [51]. The dry weight of lysed *M. aeruginosa* increased from 65.3 mg to 231 mg, whereas biomass showed no significant change during co-culture with green algae, further confirming its selective utilisation of algal cell components. Despite established evidence that hyphal attachment of *P. chrysosporium* and *T. citrinoviride* disrupts cyanobacterial membrane integrity, with *T. citrinoviride* displaying potent and specific inhibition against *M. aeruginosa*, the precise pathways of action on the cyanobacterial membrane and the ensuing cellular response have yet to be elucidated.

#### 2.2.2. Metabolite-Mediated Molecular Damage

*P. chrysosporium* [52] and *Aspergillus* species (*A. wentii*, *A. ustus*, *A. versicolor*) [53] disrupt algal physiological functions by secreting secondary metabolites (e.g., anthraquinones, terpenoids, steroids). RT-qPCR analysis revealed that *P. chrysosporium* or its metabolites significantly regulated key gene expression in *M. aeruginosa*. Relative expression levels of *prx*, *rec*A, *grp*E, and *fab*Z increased 5–10-fold compared to controls, indicating oxidative stress and DNA damage in algal cells. Conversely, *psb*D1, *rbc*L decreased by over 95%, while *mcy*B expression dropped from 5.25 ± 0.62% to 0.46 ± 0.07%, resulting in an 88.6% reduction in Chla content and impaired microcystin synthesis [52].

Among 32 pure compounds isolated from *A. wentii*, *A. ustus*, and *A. versicolor*, 11 (including 5 anthraquinones, 2 terpenoids, and 4 steroids) exhibited potent algicidal activity [53]. For *Chattonella marina*, anthraquinones exhibited a 24-h EC_50_ as low as 0.01 μg/mL, with complete cell death at 5 μg/mL. Against *Heterosigma akashiwo*, 1,5-dihydroxy-3-methoxy-7-methylanthraquinone achieved 100% inhibition at 2.5 μg/mL, reducing Chla content, increasing malondialdehyde (MDA) levels, and disrupting cell membranes and antioxidant systems.

#### 2.2.3. Dual Mechanism Driven by Photosensitization

Cercosporin (CP), a phytotoxin produced by fungi of the genus *Cercospora*, functions as a prototypical photosensitised algicide through a dual mechanism involving both reactive oxygen species (ROS) generation and direct cellular adsorption. Under visible light irradiation, 20 μM CP achieved 95% inactivation of *M. aeruginosa* within 36 h. ROS quenching experiments revealed that the superoxide anion (O_2_^−^) scavenger PBQ completely inhibited algicidal activity, whereas hydroxyl radical (•OH) and singlet oxygen (^1^O_2_) scavengers showed no significant effect, confirming O_2_^-^ as the primary active species. Lipidomic analysis revealed that CP preferentially degraded unsaturated fatty acids (e.g., C16:1, C18:1) and photosynthetic membrane lipids (e.g., sulfoquinovosyl diglyceride SQDG, phosphatidylglycerol PG), leading to increased cell membrane permeability and a 3–5-fold rise in intracellular organic matter (IOM) leakage compared to the control group. Furthermore, confocal microscopy revealed CP adsorbs onto algal cell membranes via hydrophobic interactions. Even under dark conditions, 20 μM CP reduced *M. aeruginosa* viability to 60.73%, with FCM analysis indicating 53.3% membrane damage. FTIR analysis revealed that CP’s phenolic hydroxyl groups (1204 cm^−1^, 1148 cm^−1^) embedded into algal cell wall peptidoglycan, disrupting β-(1,4)-glycosidic bonds (3350 cm^−1^) and further amplifying membrane damage effects [54].

In summary, while existing research confirms that several algicidal fungi exert significant inhibitory effects on cyanobacteria, several critical aspects require further investigation. First, the lack of specificity in some strains—which exhibit broad-spectrum inhibition against various algal species rather than targeting bloom-forming cyanobacteria exclusively—raises potential ecological concerns. Second, the precise mechanisms of action employed by algicidal fungi that specifically inhibit bloom-forming cyanobacteria remain largely ambiguous. Furthermore, challenges persist regarding the efficient isolation, cultivation, and practical large-scale deployment of these agents in environmental remediation, as evidenced by a scarcity of successful industrial application case studies.

### 2.3. Cyanophages

Cyanophages are a class of viruses that specifically infect cyanobacteria. These double-stranded DNA viruses exhibit a broad host range across numerous cyanobacterial species, thus inhibiting harmful blooms in both freshwater and marine environments, including key taxa such as *Microcystis*, *Synechococcus*, *Prochlorococcus*, and *Planktothrix* [55]. To date, approximately 250 cyanophage genomes have been deposited in the global GenBank database. However, the overwhelming majority, around 210 strains, infect marine cyanobacteria (*Prochlorococcus* and *Synechococcus*), with research on freshwater cyanophages lagging considerably. Only about 40 freshwater cyanobacterial phages have been reported, sequenced, and annotated to date. Among these, just 13 phages target *Microcystis*, including ΦMHI42, MaMV-DC, Ma-LMM01, Mic1, Ma-LBP, PhiMa05, vB_MweS-yong2, Me-ZS1, MinS1, Mae-Yong924-1, Mae-Yong1326-1, YongM, and Mwe-Yong1112-1. Among these, ΦMHI42 and Ma-LBP remain unsequenced. Only eight strains (ΦMHI42, MaMV-DC, Me-ZS1, MinS1, Mae-Yong924-1, Mae-Yong1326-1, YongM, Mwe-Yong1112-1) have been confirmed to exhibit broad-spectrum infectivity, capable of lysing hosts across cyanobacterial genera and even orders [56].

Regarding its algicidal mechanism, adsorption constitutes the initial step of phage infection, relying on the precise binding of the phage tail structure to specific receptors on the host cell surface. This process is regulated by light exposure or the host’s metabolic state in certain phages. The freshwater cyanophage AS-1 (infecting *S. elongatus* PCC6301 and PCC7942) achieved an adsorption rate of 80% within 20 min under light exposure, whereas in darkness, the adsorption rate after 20 min was only 40% [57]. Similarly, the adsorption of marine *Synechococcus* phage S-PM2 onto host WH7803 exhibits light dependency, independent of the host cell cycle. Even under synchronised host culture conditions, adsorption rates at different time points show no significant variation, confirming that light directly modulates the conformation of either the phage or host receptors, rather than indirectly influencing them via host rhythms. Furthermore, research on the broad-spectrum phage YongM revealed that its tail fibrin ORF83 is a key determinant of host range: following UV-induced mutagenesis to generate ORF83 mutant strains, this phage exhibited approximately 20% increased lysis efficiency against *Microcystis*, *Nostoc*, and other cyanobacteria by approximately 20%. It also gained the ability to infect Oscillatoriales cyanobacterial strains that the original wild-type strain could not lyse, further confirming the core role of tail structural proteins in host targeting.

Following adsorption, the phage injects its genomic DNA into the host cell, subsequently employing two strategies to achieve metabolic hijacking. Firstly, it utilises the host’s own metabolic system to synthesise viral components. Secondly, it reprograms host metabolic pathways via carried auxiliary metabolic genes (AMGs) to enhance its own replication efficiency. Cyanophage genomes commonly harbour AMGs linked to host photosynthesis, carbon metabolism, and nucleotide synthesis. For instance, the nblA gene carried by Ma-LMM01 recruits proteases to degrade host phycobiliproteins (PBS), releasing nutrients like nitrogen and sulphur for phage replication. The psbA and psbD genes carried by the *Prochlorococcus* phage P-HM2 are efficiently expressed during infection, maintaining host photosynthetic activity to provide sustained ATP and NADPH for phage replication [56]. Furthermore, studies on *Prochlorococcus* phages reveal that phage infection significantly alters host carbon metabolism: Under light conditions, the phage inhibits the host’s Calvin cycle by encoding the CP12 protein while simultaneously activating the pentose phosphate pathway. This redirects host metabolic resources to prioritise nucleotide synthesis for the phage. Conversely, in darkness, cessation of host photosynthesis causes complete halting of phage genome replication, confirming the dependency of metabolic hijacking on the host’s photosynthetic state.

Following completion of genomic replication and protein assembly within the host, the phage disrupts the host cell membrane and cell wall by encoding lysis-associated genes, releasing progeny phages and inducing host cell death. Studies on the *Planktothrix agardhii* phage PaV-LD confirm that co-expression of the endopeptidase encoded by its ORF123 gene and the membrane-associated holin protein encoded by ORF124 can achieve over 90% lysis of the host *Synechocystis* PCC6803 cells within 24 h. This lysis effect exhibits host specificity, having no impact on non-cyanobacterial microorganisms. Furthermore, significant variations in lysis efficiency were observed among different phages: the freshwater phage Lbo240-yong1 (non-*Microcystis* phage, host: *L. boryana* FACHB-240) rendered the host culture medium entirely yellow within one day, achieving rapid host clearance; Mwe-Yong1112-1 can completely lyse 7 out of 23 host strains within 3 days; whereas PhiMa05 requires 4 days for complete lysis, with an outbreak period lasting 3 days. These variations are closely related to the phage’s incubation period, burst size, and host metabolic state. Phages with short incubation periods and high burst sizes demonstrate significantly higher algicidal efficacy than strains with prolonged incubation periods and low burst sizes.

In terms of application advantages, cyanophages are naturally distributed widely, making resource acquisition readily available. Furthermore, they specifically target cyanobacteria, exerting minimal impact on non-target organisms and ecosystems, thus demonstrating excellent specificity and low ecological disturbance. Moreover, they exhibit remarkable lytic efficiency and strong environmental adaptability. Most phages maintain activity within a temperature range of 0–45 °C and a pH range of 3–10, enabling adaptation to diverse aquatic environments such as lakes and oceans. They are sensitive only to ultraviolet radiation and organic solvents, with stability further enhanced through encapsulation techniques. Additionally, synthetic biology technologies offer avenues for functional optimisation of cyanobacterial phages. However, cyanobacteria may develop resistance through genetic mutation or the CRISPR-Cas immune system, potentially necessitating frequent replenishment of new phage strains to counter resistance issues, thereby increasing application costs and complexity. Phage lysis of cyanobacteria may trigger the release of intracellular algal toxins. Should release volumes exceed the water body’s self-purification capacity, this could threaten drinking water safety and aquatic organism health. Cyanobacterial phages are also sensitive to ultraviolet radiation and organic solvents. UV light can damage their genomic DNA, while organic solvents can disrupt their protein coats, leading to rapid attenuation of phage activity in natural water bodies. Furthermore, bottlenecks persist in the synthesis and functional optimisation of engineered phages [55,56,57,58]. Future research could expand habitat screening efforts, deepen investigations into infection mechanisms, and pursue optimisation of genomic structures.

To provide a comprehensive understanding of direct algicidal microorganisms, Table 2 systematically compares the mechanisms and characteristics of different types, including algicidal bacteria, algicidal fungi and cyanophages.

## 3. Microbial Regulation of Aquatic Environments to Inhibit Cyanobacteria

### 3.1. Nitrogen-Cycling Microorganisms

Cyanobacterial proliferation is strongly coupled to the bioavailability of dissolved inorganic nitrogen (DIN). Consequently, microbial guilds involved in nitrification, denitrification, anammox and diazotrophy represent critical regulators of bloom initiation and persistence. This synthesis assesses the biochemical pathways by which nitrogen-cycling microorganisms suppress cyanobacteria, quantifies their reported field performance, and outlines their associated advantages and limitations.

#### 3.1.1. Anammox Bacteria

Research indicates that anammox primarily contributes to the control of cyanobacterial blooms indirectly by regulating the biological availability of nitrogen in aquatic systems. Its mechanism of action is closely linked to nitrogen cycling processes and responsive to environmental conditions. Firstly, anammox influences nitrogen balance as a nitrogen removal pathway. Research indicates that anammox utilises NH_4_^+^ and NO_2_^−^ to produce N_2_, converting biologically available inorganic nitrogen in the water body into gaseous nitrogen unavailable to phytoplankton. This process reduces the nitrogen sources required for cyanobacterial growth. At two sampling sites in Mississippi Bay (Parker’s River Mouth, PRM; Central Basin, MB), anammox contributed an average of 6% (PRM) and 10% (MB) to total N_2_ production, peaking at 27% (PRM, September 2009) [63]. Though subordinate to the dominant role of denitrification, this activity provides supplementary nitrogen removal services to the ecosystem, reducing nitrogen availability for cyanobacterial growth. Secondly, anammox is regulated by environmental conditions, thereby indirectly influencing dominant cyanobacterial species composition. Observations indicate that bottom-water hypoxia significantly alters anammox function: while inhibiting aerobic nitrification, it creates more favourable redox conditions for anaerobic anammox, increasing its relative contribution, which concurrently increases sediment NH_4_^+^ release. This elevates concentrations of bioavailable nitrogen species like NH_4_^+^ in the water column, while phosphorus flux remains unaffected, ultimately promoting non-nitrogen-fixing cyanobacterial blooms. Such cyanobacteria exhibit stronger competitive uptake of NH_4_^+^, enabling rapid cyanobacterial blooms under conditions of increased nitrogen supply and sufficient phosphorus. Conversely, during non-anoxic periods, anammox synergises with denitrification to continuously remove inorganic nitrogen from the water body, partially alleviating nitrogen accumulation and inhibiting excessive cyanobacterial proliferation.

#### 3.1.2. Disruption of Cyanobacterial Nitrogen Fixation

Gao et al. [64] investigated photosynthesis and nitrogen fixation during cyanobacterial blooms in the upper Susquehanna River, a tidal freshwater tributary of the Chesapeake Bay in the United States. They found that cyanobacterial mixed communities could fix carbon and nitrogen under conditions where most phytoplankton were constrained, by utilising temporal variations in dissolved inorganic carbon (DIC) and dissolved oxygen (DO). During the early cyanobacterial blooms, nitrogen limitation favoured nitrogen-fixing cyanobacteria as dominant species. Their biomass-normalised nitrogen-fixation rates and Chla-based photosynthetic rates increased with enhanced light exposure. At peak bloom, photosynthetic carbon uptake and oxygen production depleted DIC and caused oxygen supersaturation. Oxygen inhibition of nitrogenase and carbon limitation constrained photosynthesis, suppressing nitrogen fixation under light conditions, whereas dark-period fixation accounted for 40% of the daily total. Under these high pH and high dissolved oxygen conditions, cyanobacterial species known for dark-phase nitrogen fixation dominate. Research further indicates that cyanobacterial blooms constitute a vital component of nitrogen cycling in oligohaline and tidal freshwater estuaries. Their species composition and physiological adaptation strategies enable blooms to persist under adverse conditions, whilst the environmental changes driven by the bloom exert negative feedback on cyanobacterial growth.

#### 3.1.3. Integrated Management Prospects

Zhang et al. [65] isolated three mixed aerobic denitrifying bacterial communities (Mix-CADBCs: NM7, NM18, and NM39) from lake water bodies. These communities achieved removal rates exceeding 94% for both total nitrogen and dissolved organic carbon, with over 70% of initial nitrogen converted into gaseous nitrogen and less than 11% converted into microbial biomass. Batch experiments demonstrated their denitrification capability under diverse conditions, with dominant genera including *Hydrophagenia*, *Prosthecomicrobium*, and *Pseudomonas*. Network analysis revealed that *Pseudomonas* I.Bh25.14 and *Vogsella* LIG4 were associated with the removal of total nitrogen and dissolved organic carbon. When inoculated into slightly polluted lake water, they achieved a total nitrogen removal rate exceeding 78.72%, promoted microbial growth, and enhanced organic degradation capacity, offering a potential strategy for biological treatment of lake water bodies.

### 3.2. Phosphorus-Cycling Microorganisms

Phosphorus (P) exerts a primary regulatory influence on primary producers in most freshwater systems, rendering microbial-mediated phosphorus transformation pivotal in controlling cyanobacterial blooms [66]. Relevant microbial communities, including polyphosphate-accumulating bacteria (PABs), polyphosphate-degrading bacteria, and photosynthetic or chemotrophic biofilm communities, can regulate material fluxes between dissolved reactive phosphorus (DRP), particulate organic phosphorus, and mineral-bound phosphorus.

#### 3.2.1. Polyphosphate-Accumulating Bacteria

Research has revealed that PABs isolated from activated sludge can suppress cyanobacterial blooms by up to 95.56% through trapping phosphorus in the form of pyrophosphate [67]. This demonstrates that employing these microorganisms offers promising efficacy in controlling cyanobacterial blooms and, consequently, the eutrophication process.

#### 3.2.2. Periphytic Biofilms

Lu et al. [68] evaluated the impact of epibenthic biofilms on phosphorus (P) content along with its speciation at the water-sediment interface through simulated experiments. In the presence of attached biofilm, concentrations of all phosphorus species (TP, TDP, DIP, PP, and DOP) in the overlying water were significantly reduced to lower levels (<0.05 mg/L), whereas TP concentrations in the control group (without attached biofilm) increased (>1.8 mg/L). The epibenthic biofilm elevated water pH (peaking at approximately 10), promoting co-precipitation of phosphorus with metal salts and slowing the loss of phosphorus components such as Fe/Al-P and Ca-P from sediments. Furthermore, phosphorus content within the biofilm (primarily in Fe/Al-P and Ca-P forms) increased by 100% after 60 days. This indicates that epibiont biofilms can capture and store phosphorus from water, forming a buffer against release and precipitation.

#### 3.2.3. External P Control

A study investigated the influence of phosphorus concentration on cyanobacterial blooms by the development of a dynamical modelling framework [69]. This model incorporated key factors on cell growth, notably light availability and phosphorus levels, and accounted for seasonal variations in temperature and illumination. The resulting system comprised a set of periodically forced nonlinear ordinary differential equations. Findings from the study suggest that phosphorus concentration modulates the occurrence of cyanobacterial blooms by impacting algal growth rates and invasive potential. Numerical simulations further demonstrated that reducing parameters associated with internal phosphorus release decreases phosphorus concentrations and limits the invasion growth rate of algae. Furthermore, the results indicate that simultaneous reduction in external phosphorus inputs and sediment phosphorus release most effectively lowers Chla concentrations and accelerates lake ecological recovery, while controlling a single phosphorus variable produces limited effects. Beyond chemical management, manipulating photosynthetic microbial communities—such as by introducing or stimulating phototrophic organisms—can interfere with cyanobacteria’s access to light and nutrients, thus serving as a potential strategy for controlling cyanobacterial blooms.

### 3.3. Photosynthetic Bacteria

Research by Kim et al. [70] highlighted the potential of harmless phototrophic bacteria in controlling harmful cyanobacterial blooms. Their study demonstrated that *Cyanobium gracile* A950 inhibits the growth of *M. aeruginosa* by disrupting the latter’s photosynthetic efficiency and nutrient uptake. *Cyanobium gracile* A950 is characterized by its small cell size and high proliferation rates, enabling it to effectively compete for essential resources such as light, carbon, and nitrogen. This competition indirectly suppresses cyanobacterial blooms while contributing to the maintenance of ecological equilibrium.

These studies above indicate that these microorganisms do not exert a direct effect on cyanobacteria, but utilise them to control key nutrients, such as nitrogen and phosphorus, or compete for resources, like light, with cyanobacteria through beneficial microorganisms. Although they are effective environmental strategies for controlling cyanobacterial blooms, these studies are mostly in the process of laboratory research. Some of these technologies have already been applied on a small scale or in specific venues, yet the vast majority of them have not been popularised or become major control methods, lacking a grand scale and engineering application. Moreover, the effects of these bacteria are not only limited to cyanobacteria, but also have inhibitory effects on other algae. So their functions are broad-spectrum, and thus their ecological risks also need to be further evaluated.

## 4. Combined Applications of Microorganisms with Other Technologies

### 4.1. Microbial + Biofilm Technology

The integration of microbial treatment with biofilm systems constitutes an effective strategy for controlling cyanobacterial blooms. Biofilms provide a stable microhabitat for beneficial microorganisms, sustaining their activity while removing nutrients to inhibit algal growth.

Lu et al. [68] demonstrated that epiphytic biofilms formed on submerged surfaces, such as stones, plant roots, or artificial substrates, can adsorb and immobilise phosphorus at the sediment-water interface. This buffering effect reduces internal phosphorus loading and prevents eutrophication.

Based on this, Liang et al. [71] reviewed the design, construction, and optimisation of mixed-species microalgal biofilms in wastewater treatment, particularly their capacity to remove nutrients and suppress harmful algae. They emphasised selecting compatible photosynthetic and heterotrophic species and optimising surface attachment characteristics. Such biofilms may comprise algae, cyanobacteria, bacteria, and fungi coexisting within communities to perform multiple ecological functions.

In practice, integrating algicidal microorganisms with biofilm scaffolds creates localised microenvironments that simultaneously capture nutrients and effectively suppress cyanobacteria. For instance, algicidal bacteria attached to biofilms can produce extracellular enzymes, reactive oxygen species, or allelochemicals. These substances diffuse into the surrounding water body, acting upon species responsible for algal blooms. Simultaneously, the biofilm matrix enhances microbial retention, shielding them from environmental stresses and thereby improving treatment efficacy.

Moreover, biofilm-associated systems enable multifunctional interventions, including nutrient uptake, algal dissolution, and pollutant degradation. Such systems are particularly suited for large-scale applications in constructed wetlands, retention ponds, or floating island ecosystems, facilitating sustainable ecosystem restoration.

### 4.2. Microbial + Phytoremediation

The integration of microbial agents with aquatic plants, known as microbial phytoremediation technology, offers an ecologically sound strategy for mitigating harmful cyanobacterial blooms. This method utilises both the nutrient uptake capacity and allelopathic properties of large aquatic plants, whilst leveraging the metabolic diversity advantages of associated microbial communities.

Multiple aquatic plants have been demonstrated to exert direct inhibitory effects on cyanobacteria. For instance, Zhang et al. [72] observed that *Cyperus rotundus* root extract significantly reduced *Microcystis* biomass within experimental ponds over 30 days, indicating potent allelopathic activity.

Beyond direct allelopathic effects, aquatic plants play a pivotal role in removing excess nutrients such as nitrogen and phosphorus, thereby limiting the substrate required for algal bloom growth. They are ideal carriers for nutrient recovery in eutrophic water bodies.

Plant-microbe consortia are particularly suited for engineered ecological infrastructure like man-made wetlands, floating treatment ponds, or floating gardens. Such systems can be tailored to specific microbial-plant combinations to maximise nutrient cycling and algicidal activity. For instance, Li and Pan [73] developed a system for Lake Tai that integrated chitosan with *Pseudomonas* strains within a soil-covered substrate, achieving simultaneous cyanobacterial removal and microcystin degradation, thereby demonstrating the feasibility of microbial phytoremediation techniques. Furthermore, this biohybrid system exhibits long-term ecological stability, low energy consumption, and holds promising development prospects.

### 4.3. Microbial + Physical/Chemical Methods

Integrating microbial strategies with physical and chemical methods represents a cutting-edge approach to controlling cyanobacterial blooms. This methodology leverages the complementary advantages of diverse mechanisms, including oxidation, adsorption, and biodegradation, to achieve highly efficient treatment.

A notable example is the enhanced constructed wetland system utilising beneficial microorganisms. The microcosm wetland system developed by Wang et al. [74] significantly improved the removal efficiency of *M. aeruginosa* and its associated toxin microcystin-LR by establishing engineered microbial communities. This approach not only degrades toxins but also removes nutrients.

In another innovative study, Nam et al. [75] employed chitosan-coated oxygen nanobubbles to control cyanobacteria. The oxidative stress induced by these nanobubbles selectively inactivated approximately 73% of *Microcystis* cells without triggering explosive cell lysis, which could release substantial amounts of intracellular toxins. The chitosan coating not only enhanced the stability of the nanobubbles but also enabled the controlled, sustained release of reactive oxygen species.

These examples demonstrate that multimodal interventions combining biological specificity with physical or chemical efficacy can overcome the limitations of single approaches. For instance, while photocatalysis enables rapid inactivation, microbial biofilms ensure long-term nutrient cycling and ecological equilibrium. Similarly, chemical agents like nano-bubbles initiate algal cell disruption, subsequently releasing organic compounds through microbial degradation.

Moreover, such integrated systems permit customised modular solutions applicable across diverse aquatic environments, including lakes, aquaculture ponds, and treatment wetlands. The flexible combination of microbial preparations with catalysts, adsorbents, or oxidative nanomaterials opens new avenues for tailored, environmentally responsive harmful algal bloom control technologies.

To better illustrate the advantages and disadvantages of the various microorganism-based strategies for controlling cyanobacterial blooms mentioned above, Table 3 provides a comparative overview of their strengths, limitations, typical application cases, and applicable water bodies.

## 5. Conclusions and Future Prospects

In summary, significant progress has been achieved in multiple microbial strategies for controlling cyanobacterial blooms. Directly algicidal microorganisms, including bacteria, fungi, and bacteriophages, exert a lytic effect on harmful cyanobacteria, effectively reducing bloom biomass. Concurrently, other microbial communities indirectly suppress bloom development by influencing key environmental factors such as nutrient cycling, light conditions, and ecosystem interactions. The integration of microbial preparations with biofilms, aquatic plants, and physicochemical techniques has demonstrated synergistic enhancement in control efficacy. Although these approaches possess distinct advantages and limitations, their combined application, such as coupling bioaugmentation with nutrient removal and habitat modification, holds promise for achieving comprehensive and sustainable algal bloom management. Table 4 outlines the research focuses, core concepts, advantages, challenges, and development trends in this field for gaining a deeper insight into the future development landscape of microbial control for cyanobacterial blooms.

Looking ahead, research should focus on advancing cross-disciplinary collaboration and technological innovation to optimise microbial control strategies. Advances in genomics, synthetic biology, materials science, and ecological modelling present promising opportunities for refining these approaches. For instance: enhancing control efficacy and stability through engineered cyanophage specificity, developing potent algicidal enzymes, and designing functionally complementary microbial communities. Furthermore, intelligent delivery systems such as microencapsulation and nanocarriers enable targeted precision application in aquatic environments. Deep understanding of algal bloom formation mechanisms under varying environmental conditions is crucial for implementing precise interventions. Continuous monitoring of ecological impacts and public health safety is key to ensuring microbial control methods are both effective and environmentally sound. Through sustained cross-disciplinary collaboration, microbial-based strategies hold promise as an integral component of global cyanobacterial blooms management, contributing to the restoration and conservation of freshwater ecosystems.

## Figures and Tables

**Table 1:** Summary of Algicidal Bacteria and Their Targeted Cyanobacterial Species.

Algicidal Bacteria	Target Algal Species	Mechanism (Certainty Level)	Algicidal Efficacy/Extent	Reference
*Aeromonas*	*Microcystis aeruginosa*, *Microcystis flos-aquae*, *Anabaena cylindrica*, *Dolichospermum flos-aquae*, *Nodularia spumigena*	Direct contact-dependent lysis via intracellular protease-like substances; disrupts cell structure (High)	91% lysis of *M. aeruginosa*; extremely low activity against non-target algae	[15,16]
*Alcaligenes*	*Microcystis aeruginosa*	Disrupts cell membranes, photosynthetic lamellae, and phycobiliproteins (Medium)	Algicidal rate: 74.41% (high algal density) to 88.45% (low algal density); wheat bran-supplemented immobilization enhances efficacy to 95.49%	[17,18]
*Arthrobacter*	*Microcystis aeruginosa*	Presumed secretion of algicidal metabolites (Low; mechanism not elaborated)	Inhibits growth of *M. aeruginosa*; no quantitative efficacy provided	[19]
*Bacillus*	*Microcystis aeruginosa*, *Aphanizomenon gracile*, *Aphanizomenon flos-aquae*, *Microcystis viridis*, *Microcystis wesenbergi*, *Oscillatoria tenuis*, *Nostoc punctiforme*, *Dolichospermum flos-aquae*, *Limnospira maxima*	Secretes enzymes/metabolites that disrupt cell walls/membranes; direct lysis (High)	Reduces cyanobacterial density from 600,000 to 80,000 cells/mL; eliminates harmful algae in Dianchi Lake; high efficacy against diverse cyanobacteria	[18,20,21,22]
*Bdellovibrio*	*Microcystis aeruginosa*	Obligate endosymbiotic predation; enzyme-mediated degradation of host cellular components (High)	Lyses *M. aeruginosa* and *Phormidium luridum*; invades host cells for internal lysis	[22,23,24]
*Brevibacillus*	*Microcystis aeruginosa*	Presumed secretion of algicidal active substances (Low; mechanism not elaborated)	Inhibits growth of *M. aeruginosa*	[25]
*Burkholderia*	*Microcystis aeruginosa*	Biodegradation of microcystins; indirect growth inhibition (Medium)	Suppresses *M. aeruginosa* proliferation; degrades cyanotoxins	[26]
*Chryseobacterium*	*Microcystis aeruginosa*	Secretes heat-stable extracellular substances; disrupts cell membranes and photosynthetic systems (High)	72-h algicidal rate: 80%; time-dependent lysis (35% at 24 h, 62% at 48 h)	[27]
*Cytophaga*	*Nostoc muscorum*, *Leptolyngbya boryana*	Direct cell lysis (Medium)	Lyses target cyanobacterial cells	[28]
*Exiguobacterium*	*Microcystis aeruginosa*	Secretes glycolipid-like substances; damages cell walls/membranes and inhibits chlorophyll synthesis (Medium)	Highly effective against *M. aeruginosa* 7820; reduces intracellular pigment content	[29]
*Flavobacterium*	*Microcystis* sp., *Anabaena* sp.	Presumed secretion of algicidal metabolites (Low; mechanism not elaborated)	Inhibits the growth of target cyanobacteria	[30]
*Flexibacter*	*Oscillatoria williamsii*	Direct cell lysis (Medium)	Lyses *O. williamsii*	[13]
*Lysinibacillus*	*Microcystis aeruginosa*	Presumed secretion of algicidal substances (Low; mechanism not elaborated)	Inhibits growth of *M. aeruginosa*	[31]
*Microbacterium*	*Microcystis aeruginosa*	Presumed algicidal activity (Low; mechanism not elaborated)	Inhibits growth of *M. aeruginosa*	[32]
*Morganella*	*Microcystis aeruginosa*, *Aphanizomenon gracile*	Induces molecular stress response leading to cell death (Medium)	Suppresses growth of target cyanobacteria	[20]
*Myxococcus*	*Anabaena*, *Microcystis*, *Oscillatoria*, *Phormidium*	Extracellular predation; collective secretion of hydrolytic enzymes (High)	Captures and lyses diverse cyanobacteria via aqueous colonies	[13,22]
*Nocardioides*	*Microcystis aeruginosa*	Direct physical attack; adheres to cell surfaces, disrupts structure, and downregulates photosynthetic/toxin-related genes (High)	1-day algicidal rate: 84%; 2-day rate: 98%; specific to *M. aeruginosa* (no impact on most non-target algae)	[33]
*Paenibacillus*	*Microcystis aeruginosa*	Secretes extracellular active substances; disrupts cell membranes and cell walls (High)	Algicidal rate: 89.51–90.48% against *M. aeruginosa*; potent against cyanobacteria, minimal impact on green algae	[34]
*Pseudoalteromonas*	*Microcystis aeruginosa*	Presumed algicidal activity (Low; mechanism not elaborated)	Inhibits growth of *M. aeruginosa*	[15,35]
*Pseudomonas*	*Microcystis aeruginosa*	Secretes enzymes/secondary metabolites; disrupts cell walls/membranes (High)	Maximum algicidal effect (MAE) from −17.4% to 83.2% (mesophilic/cold conditions); no efficacy in flowing water	[36]
*Raoultella*	*Microcystis aeruginosa*	Damages photosynthetic systems; inhibits carbohydrate metabolism and DNA replication/repair (High)	72-h kill rate from 37.21% (high algal density) to 96.23% (low algal density); induces flocculation	[37,38]
*Rhizobium*	*Microcystis aeruginosa*	Biodegradation of microcystin-LR via mlrA gene (Medium)	Inhibits *M. aeruginosa* growth; degrades cyanotoxins	[39]
*Saprospira*	*Anabaena* sp.	Forms bundles to lyse cyanobacterial cells (Medium)	Lyses *Anabaena* sp.	[40]
*Serratia*	*Microcystis aeruginosa*	Synthesizes serratiamycin; dose-dependent algicidal activity (High)	3.0 μg/L serratiamycin achieves 91.1% algicidal rate	[41,42]
*Shewanella*	*Microcystis aeruginosa*	Presumed algicidal activity (Low; mechanism not elaborated)	Inhibits growth of *M. aeruginosa*	[43]
*Sphingomonas*	*Microcystis, Anabaena*	Presumed secretion of algicidal metabolites (Low; mechanism not elaborated)	Inhibits growth of target cyanobacteria	[30]
*Staphylococcus*	*Microcystis aeruginosa*	Presumed algicidal activity (Low; mechanism not elaborated)	Inhibits growth of *M. aeruginosa*	[32]
*Stappia*	*Microcystis aeruginosa*	Presumed algicidal activity (Low; mechanism not elaborated)	Inhibits growth of *M. aeruginosa*	[32]
*Streptomyces*	*Microcystis aeruginosa*, *Dolichospermum* sp., *Pseudanabaena* sp., *Anabaena* sp., *Synechocystis* sp.	Secretes algicidal active substances (Medium)	Inhibits growth of diverse cyanobacteria	[13,44]
*Vibrio*	*Oscillatoria amphibia*	Produces β-cyanoalanine; specific inhibition (Medium)	Inhibits growth of *O. amphibia*	[45]

**Table 2:** Comparative Mechanisms and Characteristics of Different Direct Algicidal Microorganisms.

Microorganism Type	Representative Genera/Species	Algicidal Mechanism	Advantages	Limitations	Reference
**Algicidal Bacteria**	*Bacillus*, *Pseudomonas*, *Aeromonas*, *Raoultella*	Secrete hydrolytic enzymes, reactive oxygen species (ROS), organic acids, or lipopeptides to lyse cyanobacterial cells	Fast-acting; some strains are non-toxic; environmentally friendly; can degrade cyanotoxins	Susceptible to environmental conditions; competition with indigenous microbes; short survival in open waters	[59,60,61,62]
**Algicidal Fungi**	*Phanerochaete chrysosporium*, *Trichoderma citrinoviride*	Induce oxidative stress; disrupt photosynthetic gene expression; produce secondary metabolites (e.g., cercosporin)	Can simultaneously inhibit growth and degrade toxins; effective even in low concentrations	Growth rate slower than bacteria; less studied; ecological safety needs assessment	[52,53,54]
**Cyanophages (Cyanobacterial Viruses)**	Cyanophages infecting *Microcystis*, *Synechococcus*, etc.	Adsorption, penetration, replication, lysis of host cells (via phage-induced cell rupture)	High specificity; can be engineered for targeted control; low ecological footprint	Host-specificity limits spectrum; ecological interactions complex; still under development for field use	[56,58]

**Table 3:** Comparative Advantages and Limitations of Various Microbial-Based Cyanobacterial Bloom Control Strategies.

Strategy Type	Specific Strategy	Advantages	Limitations	Typical Application Cases	Suitable Water Bodies	Reference
Direct Algicidal Microorganisms	Algicidal bacteria (*Bacillus*, *Pseudomonas*)	Rapid cell lysis; eco-friendly; toxin degradation	Sensitive to environmental changes; short persistence	*Bacillus cereus* lysed *Microcystis*	Freshwater lakes, reservoirs	[14,15,21,37]
	Algicidal fungi (*Phanerochaete*, *Trichoderma*)	Oxidative stress induction; toxin degradation; metabolite secretion	Slower action; limited field data	*Phanerochaete chrysosporium* suppressed *Microcystis*	Shallow lakes, ponds	[50,51,52]
	Cyanophages	High specificity; self-replicating; low ecological footprint	Host specificity; possible resistance	Ma-LMM01 infects *Microcystis*	Mesocosms; potential field use	[55,56,57,58]
Indirect Microbial Regulation	Nitrogen cycle manipulation (denitrifiers)	Nitrogen depletion limits bloom resurgence	Possible N_2_O release; microbial competition	*P. stutzeri* improved denitrification	Eutrophic lakes, rivers	[63,65]
	Phosphorus cycling microbes	Reduce soluble P via adsorption/precipitation	Re-release under anoxia; variable effectiveness	*Bacillus* strains mineralized and absorbed P	Wetlands, shallow lakes	[66,67,73]
Microbial-Plant Synergy	Microbial-macrophyte consortia	Synergistic nutrient uptake; microbial shelter	Seasonality; ecological complexity	*Chlorella*-bacteria removed N, P, and inhibited blooms	Constructed wetlands, buffer zones	[71,74]
Microbial + Physicochemical	Microbes + flocculants/clays/UV	Fast-acting; synergistic effects; multi-target removal	Additive cost and safety; microbial viability risk	Bacteria + Phoslock removed P	Emergency bloom control in lakes	[66,73,75]
Biofilm Reactor Systems	Floating/fixed microbial biofilms	Long-term action; stable; reusable systems	Biofilm detachment; engineering complexity	Biofilms reduced *Microcystis*	Canals, low-flow eutrophic waters	[68,71]

**Table 4:** Research Focus and Development Trend in Microbial Control of Cyanobacterial Blooms.

Research Focus	Core Concept	Advantages and Innovations	Challenges and Open Questions	Development Trend	Reference
Synthetic/Engineered Microbes	Use of synthetic biology to design custom microbes with enhanced algicidal or nutrient-transforming abilities	Target specificity; multi-functionality; toxin degradation; controllable traits	Environmental release risk; gene stability; biosafety regulation	Rapid development; field trials increasing	[55,56,62]
Smart Delivery Systems	Microcapsules, hydrogels, nanocarriers to deliver microbial agents or enzymes	Improved stability and survivability; targeted release; reduced environmental impact	Material compatibility; scale-up cost; release kinetics control	Applied in precision treatment strategies	[18,73,75]
Multi-omics Tools	Integration of metagenomics, transcriptomics, proteomics, metabolomics	Mechanistic insights; identification of novel functional strains and pathways	Data integration complexity; expensive infrastructure; interpretation challenges	Becoming standard in microbial ecology	[2,34,48,70]
Ecological Safety Assessment	Long-term monitoring of microbial intervention effects on biodiversity and ecosystem functions	Ensures environmental sustainability and public acceptance	Time-consuming; standard evaluation metrics still evolving	Policy-driven demand increasing	[62,63,64]
Microbial Consortia Design	Constructing synergistic microbial communities for stable and resilient bloom suppression	Increased ecological stability; better resistance to environmental variability	Community dynamics hard to predict; interspecies competition	Moving from mono- to multi-strain applications	[18,31,34,71,74]
Hybrid Integrated Approaches	Coupling microbial methods with physical, chemical, or ecological tools	Synergistic effect; emergency responsiveness; multiple inhibition paths	System integration; risk of overcomplication	Widely adopted in practical water management	[66,73,74,75]
AI and Modeling Support	Using machine learning and ecological modeling to predict outcomes and optimize strategies	Predictive deployment; data-driven optimization; reduced field testing burden	Model generalization; requires large datasets	Early-stage, with growing interest	[2,46,69,71]

## Data Availability

The original contributions presented in this study are included in the article. Further inquiries can be directed to the corresponding authors.
